# Chronic Plaque Psoriasis With Persistent Facial Involvement Successfully Treated With Risankizumab: A Case Report

**DOI:** 10.7759/cureus.101702

**Published:** 2026-01-16

**Authors:** Reem AlQusaimi, Fawziah AlRujaib, Fahad AlSharhan, Suad Alassaf, Layal Khoursheed, Fatemah Abdulrahman, Doaa AlAwadhi

**Affiliations:** 1 Dermatology, Abdulkareem Al-Saeed Dermatology Center, Kuwait City, KWT; 2 Dermatology, Kuwait Institute for Medical Specializations, Kuwait City, KWT; 3 Medicine and Surgery, Jaber Al-Ahmed Hospital, Kuwait City, KWT; 4 Dermatology, Mussaed Al-Saleh Health Center, Kuwait City, KWT

**Keywords:** chronic plaque psoriasis, facial psoriasis, risankizumab, site-specific treatment response, targeted immunotherapy

## Abstract

Chronic plaque psoriasis is a common immune-mediated inflammatory skin disease; however, facial involvement is relatively uncommon and may present diagnostic and therapeutic challenges. Facial psoriasis can mimic other inflammatory dermatoses and may demonstrate variable responses to systemic therapy. We report a case of a 37-year-old male with chronic plaque psoriasis who presented with extensive plaques involving the elbows and knees, along with diffuse, generalized facial erythema. The patient had severe disease with extensive body surface area involvement, reflected by a Psoriasis Area and Severity Index (PASI) score of approximately 50. Histopathological examination of skin punch biopsies confirmed the diagnosis of psoriasis. Initial treatment with systemic cyclosporine resulted in partial improvement. Due to persistent disease activity and the need for long-term control, therapy was escalated to risankizumab, leading to near-complete systemic clearance. Despite excellent overall disease control, residual facial erythema persisted and was managed with topical therapy and adjunctive pulsed dye laser treatment, selected to target persistent vascular erythema. This case highlights the site-specific variability in treatment response observed in facial psoriasis and underscores the importance of individualized, multimodal management strategies to optimize both clinical and psychosocial outcomes.

## Introduction

Psoriasis is a chronic, immune-mediated inflammatory skin disease affecting approximately 2-3% of the global population. Chronic plaque psoriasis is the most common clinical subtype and is characterized by well-demarcated erythematous plaques with silvery scale, most frequently involving the scalp, trunk, and extensor surfaces. Disease pathogenesis is driven by dysregulated interactions between innate and adaptive immune pathways, with a central role played by the interleukin (IL)-23/IL-17 axis [1].

Facial involvement in psoriasis is relatively uncommon compared with other anatomical sites but is clinically significant due to its visibility and disproportionate impact on quality of life. Facial psoriasis has been described in distinct phenotypic patterns, including peripheral (hairline and temples), centrofacial (cheeks, perioral, and nasolabial areas), and mixed distributions. Clinically, facial psoriasis often presents with erythema and minimal scaling, frequently mimicking other inflammatory dermatoses such as seborrheic dermatitis or rosacea, which may delay diagnosis [2,3].

In addition to diagnostic challenges, facial psoriasis may demonstrate variable or incomplete responses to systemic therapies. Proposed explanations for this site-specific behavior include differences in skin barrier function, vascular density, local immune activity, and heightened exposure to environmental triggers, all of which may influence treatment response. Despite the high efficacy of IL-23-targeted biologic therapies in achieving systemic disease clearance, persistent facial involvement has been reported and remains incompletely understood [1].

Herein, we report a case of chronic plaque psoriasis with predominant and persistent facial involvement, confirmed histopathologically, which demonstrated an excellent systemic response to risankizumab, with residual facial erythema persisting at the four-month follow-up and requiring adjunctive therapy. This case highlights the diagnostic complexity and site-specific therapeutic challenges of facial psoriasis and underscores the importance of individualized management strategies.

## Case presentation

**Figure 1:**
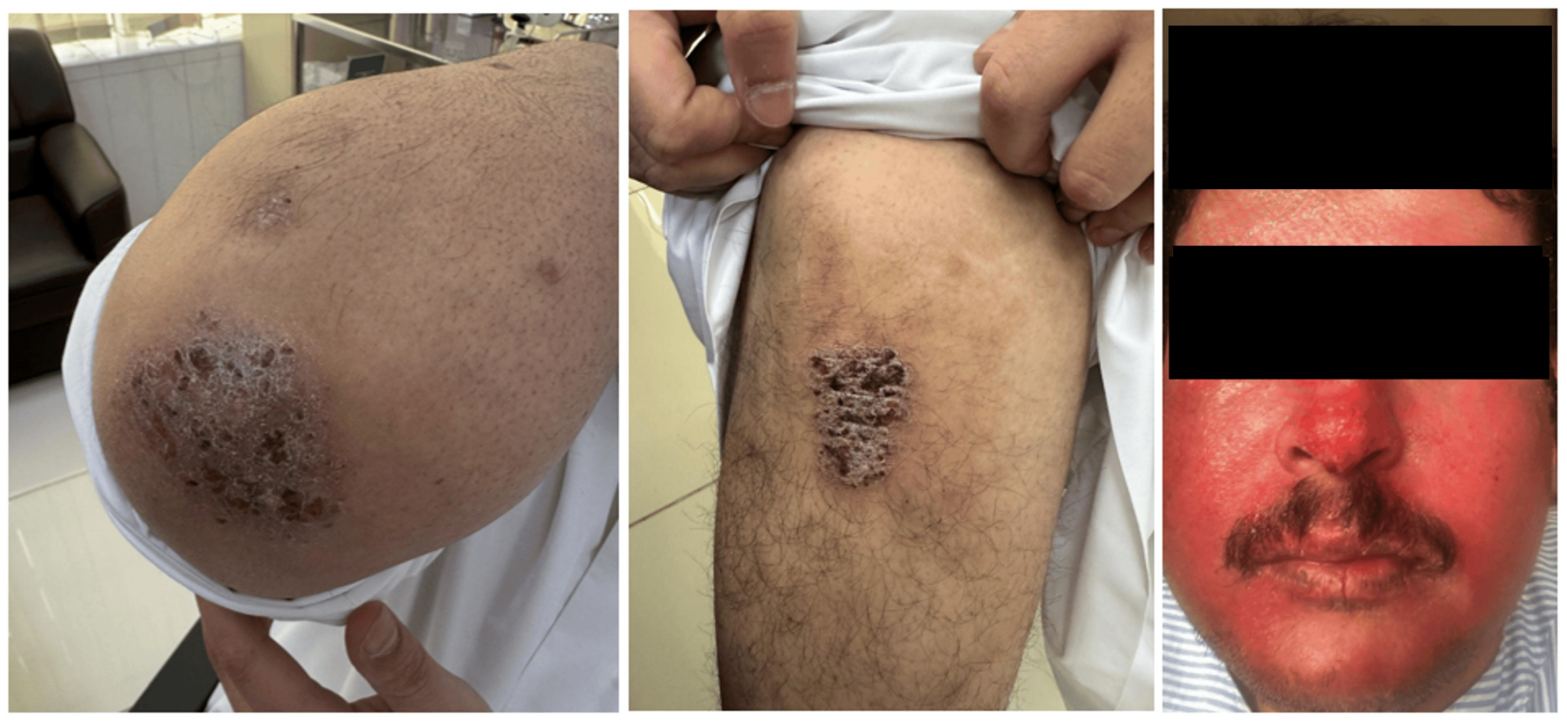
Cutaneous findings at initial presentation in chronic plaque psoriasis. Clinical photographs demonstrating active psoriatic plaques involving the elbows and knees, characterized by well-demarcated erythema and scaling. Facial involvement is noted as diffuse, generalized erythema affecting the entire face in a mask-like distribution, with minimal scaling, consistent with extensive facial psoriasis at initial presentation.

A 37-year-old male, a known case of chronic plaque psoriasis, initially presented with active psoriatic lesions involving the elbows, knees, and face for approximately four years, associated with erythema, scaling, and pruritus (Figure 1). Cutaneous involvement was extensive, affecting an estimated >30% of body surface area (BSA), with marked erythema and plaque thickness, resulting in a Psoriasis Area and Severity Index (PASI) score [4] of approximately 50, consistent with severe disease. Facial involvement was characterized by diffuse, generalized erythema in a mask-like distribution with minimal scaling, consistent with a centrofacial-predominant pattern.

**Figure 2:**
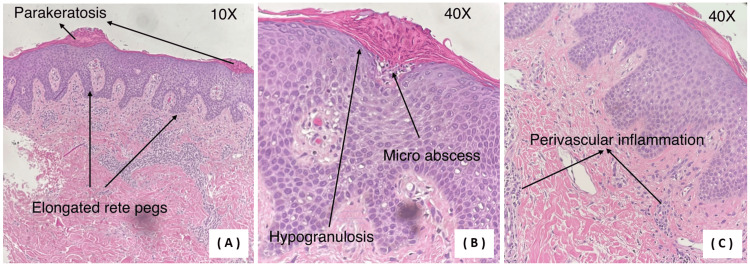
Histopathological features of psoriasis (hematoxylin and eosin stain). (A) Low-power view of a skin punch biopsy showing psoriasiform epidermal hyperplasia characterized by regular acanthosis with uniform elongation of rete ridges (test-tube-shaped rete pegs). Parakeratosis is present in the stratum corneum with associated hypogranulosis, and the superficial dermis demonstrates a mild inflammatory infiltrate. (B) High-power view of the epidermis demonstrating marked hypogranulosis with thinning to absence of the granular layer and persistent parakeratosis, reflecting abnormal keratinocyte maturation. Neutrophilic collections within the stratum corneum consistent with Munro microabscesses are present, with occasional spongiform pustules of Kogoj identified within the epidermis. (C) High-power view of the papillary dermis demonstrating a superficial perivascular lymphocytic infiltrate with mild dilation of dermal capillaries. No evidence of vasculitis or deep dermal inflammatory infiltrate is identified, consistent with the immune-mediated inflammatory pattern of psoriasis.

Based on the clinical presentation, a skin punch biopsy obtained from the face and right arm was performed, and confirmed the diagnosis of psoriasis (Figure 2). Given the atypical facial involvement, the patient was managed as psoriasis presenting with predominant facial erythema in addition to chronic plaque psoriasis. Prior to initiation of systemic therapy, a comprehensive baseline evaluation was undertaken. Laboratory investigations, including complete blood count (CBC), renal function tests (RFT), liver function tests (LFT), and metabolic profile, were all within normal limits (Table 1). Serologic screening for viral infections, including hepatitis B, hepatitis C, and human immunodeficiency virus (HIV), was negative (Table 2).

**Table 1:** Baseline laboratory investigations prior to initiation of systemic therapy. Baseline laboratory investigations were performed prior to the initiation of systemic therapy, including complete blood count, renal function tests, liver function tests, and metabolic profile. All measured parameters were within normal reference ranges. WBC: white blood cells; RBC: red blood cells; ALT: alanine transaminase; AST: aspartate aminotransferase.

Test	Value	Reference range (unit)
WBC	9.2	4 – 10 (10^9^/L)
RBC	4	3.8 – 4.8 (10^12^/L)
Hemoglobin	148	120 – 150 (g/L)
Platelet count	309	150 – 410 (10^9^/L)
Lymphocytes	2.5	1 – 3 (10^9^/L)
Creatinine	66	49 – 90 (umol/L)
Sodium	138	136 – 144 (mmol/L)
Potassium	4.4	3.6 – 5.1 (mmol/L)
Total protein	68	66 – 83 (g/L)
Albumin	40	35 – 52 (g/L)
Total bilirubin	10.2	5 – 21 (umol/L)
ALT	31	3 – 35 (U/L)
AST	21	3 – 35 (U/L)
Total cholesterol	4.8	3 – 5.2 (mmol/L)
Triglyceride	1.63	0.4 – 1.75 (mmol/L)

**Table 2:** Viral serology screening prior to systemic therapy. Summary of baseline viral serologic screening, including hepatitis B virus, hepatitis C virus, and human immunodeficiency virus, obtained before initiation of systemic immunosuppressive therapy. All tests yielded negative results. HBsAg: hepatitis B surface antigen; Anti-HCV: anti-hepatitis C virus; Ag/Ab: antigen/antibody.

Virology	
Test	Results
HBsAg in blood	Non-reactive
Anti-HCV in blood	Non-reactive
HIV Ag/Ab in blood	Non-reactive

Prior treatment history included high-potency topical corticosteroids, vitamin D analogues, and topical calcineurin inhibitors, with inadequate disease control. Phototherapy had not been initiated due to extensive facial involvement and patient preference. Given the extent of cutaneous involvement and insufficient response to topical therapy alone, the patient was initiated on systemic cyclosporine (Neoral) at a dose of 100 mg twice daily (approximately 2.5 mg/kg/day). This was combined with topical therapies, including calcipotriol/betamethasone dipropionate (Daivobet) and tacrolimus ointment (Protopic) for body lesions, and tacrolimus or pimecrolimus (Elidel) for facial involvement, depending on availability. Partial clinical improvement was observed, predominantly in body lesions; however, facial psoriasis remained active.

Due to persistent disease activity and the need for sustained long-term disease control, treatment was escalated to biologic therapy with risankizumab (Skyrizi). The patient received induction dosing according to the recommended protocol, while cyclosporine was gradually tapered, with close monitoring of blood pressure and renal function.

**Figure 3:**
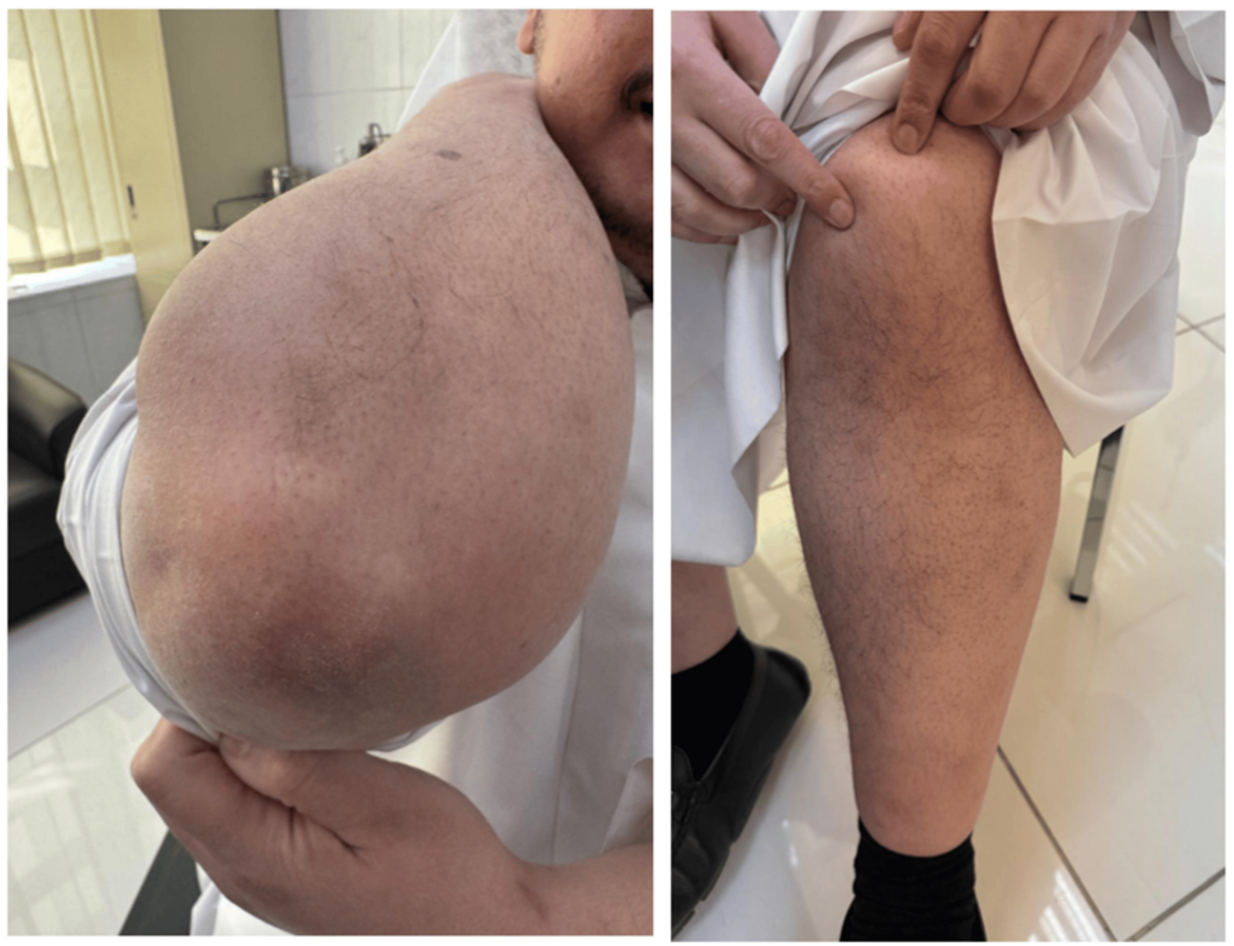
Clinical response to risankizumab at four-month follow-up. Representative clinical images of the elbows and knees at four months after initiation of risankizumab showing marked clinical improvement with near-complete resolution of psoriatic plaques, erythema, and scaling.

Following initiation of risankizumab, the patient demonstrated marked clinical improvement, with near-complete clearance of body lesions, resolution of pruritus, and significant reduction in erythema and scaling. Subsequent assessments showed a PASI improvement of approximately 90, with no active psoriatic lesions on the trunk or extremities (Figure 3).

**Figure 4:**
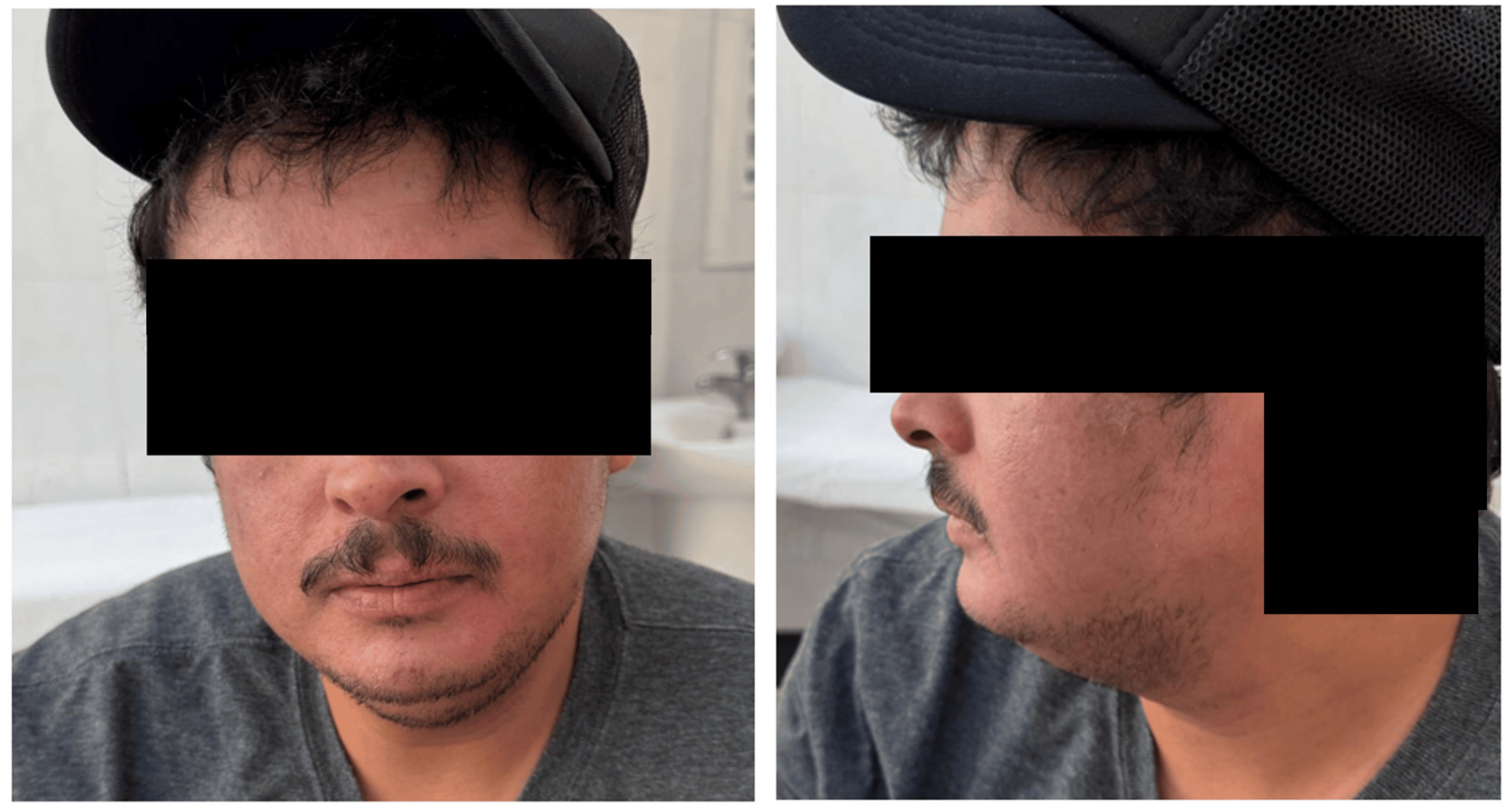
Partial resolution of facial psoriasis at four-month follow-up. Clinical photographs of the face obtained at the four-month follow-up demonstrate significant improvement with marked reduction of diffuse facial erythema, with mild residual erythema representing the only site of ongoing disease activity despite excellent systemic disease control with risankizumab.

Despite excellent systemic disease control, residual facial erythema persisted at the four-month follow-up as the only site of ongoing activity (Figure 4). This was managed with topical pimecrolimus cream, and due to recurrent facial flares, adjunctive pulsed dye laser (PDL) therapy was initiated, consisting of multiple sessions targeting persistent vascular erythema.

At the most recent follow-up, the patient remains stable on risankizumab 150 mg administered every 12 weeks. Treatment has been well-tolerated, with no reported adverse effects. Patient-reported outcomes improved substantially, with marked improvement in pruritus and quality of life, and disease control remains optimal, with no active body lesions and only minimal residual facial erythema.

## Discussion

Facial involvement in psoriasis represents a distinct clinical phenotype that may occur as part of generalized plaque psoriasis or as a predominant site of disease. Clinically, facial psoriasis often presents with erythema and subtle scaling, frequently involving centrofacial or peripherofacial regions, and may resemble other inflammatory dermatoses such as seborrheic dermatitis or rosacea. Despite limited body surface area involvement, facial psoriasis is associated with a disproportionate psychosocial burden and may exhibit variable responses to systemic therapies, underscoring its clinical relevance [5].

Although IL-23 inhibitors, including risankizumab, have demonstrated high efficacy and durable disease control in moderate-to-severe plaque psoriasis, data specifically addressing treatment response at facial sites remain limited. Large phase III clinical trials primarily assess global disease severity using composite indices such as the PASI and do not routinely report outcomes by anatomical site. Emerging clinical experience suggests that psoriasis involving special sites, including the face, scalp, and nails, may demonstrate delayed or incomplete responses despite excellent systemic clearance. The present case is consistent with these observations, with near-complete resolution of truncal and extremity lesions contrasted by persistent facial erythema [6].

The pathophysiology underlying site-specific persistence of facial psoriasis is likely multifactorial. In addition to differences in epidermal barrier function and local immune cell composition, facial skin is characterized by increased vascular density and heightened neurovascular innervation. Neurogenic inflammation mediated by neuropeptides such as substance P and calcitonin gene-related peptide may promote vasodilation, erythema, and sustained local inflammation. This neurovascular dysregulation may contribute to the persistence of facial erythema even in the setting of effective systemic cytokine inhibition, providing a biological explanation for the differential treatment response observed in facial psoriasis [5].

Adjunctive therapies targeting localized inflammation and vascular components may therefore be beneficial in selected cases. PDL therapy has been shown to improve persistent erythema in inflammatory dermatoses by selectively targeting superficial dermal vasculature and modulating inflammatory signaling. In the present case, PDL was employed as an adjunct to systemic IL-23 inhibition to address residual facial erythema following marked systemic disease control. This highlights the value of a multimodal, site-specific treatment approach in managing facial psoriasis [7].

## Conclusions

Facial involvement in chronic plaque psoriasis represents a distinct diagnostic and therapeutic challenge due to its atypical presentation and variable response to systemic therapy. This case demonstrates that while interleukin-23 inhibition with risankizumab can achieve excellent overall disease control, residual facial disease may persist and require adjunctive localized treatment. Early recognition of facial psoriasis, supported by histopathological confirmation, is essential to avoid misdiagnosis and treatment delay. An individualized, multimodal management approach is crucial to optimize both clinical outcomes and quality of life in patients with site-specific psoriasis involvement.
